# Case Report: Posterior Thoracic Window in the Presence of Pleural Effusion in Critical Care Medicine: One More Chance to Image the Aortic Valve

**DOI:** 10.3389/fcvm.2022.907168

**Published:** 2022-06-23

**Authors:** Francesca Mantovani, Giovanni Benfari, Andrea Barbieri, Francesco Manca, Vincenzo Guiducci, Alessandro Navazio, Marie-Annick Clavel

**Affiliations:** ^1^Department of Cardiology, Azienda USL, IRCCS di Reggio Emilia, Reggio Emilia, Italy; ^2^Section of Cardiology, Department of Medicine, University of Verona, Verona, Italy; ^3^Department of Cardiology, Policlinico Hospital, Modena and Reggio Emilia University, Modena, Italy; ^4^Institut Universitaire de Cardiologie et de Pneumologie de Québec (Quebec Heart and Lung Institute), Université Laval, Québec, QC, Canada

**Keywords:** case report, posterior thoracic view, aortic stenosis, aortic regurgitation, echocardiography

## Abstract

Good quality echocardiographic images in the setting of critical care medicine may be difficult to obtain for many reasons. We present a case of an 85-year-old woman with acute pulmonary edema and pleural effusion, where transthoracic bedside echocardiographic examination raised a suspicion for significant aortic valve disease. However, given the orthopneic decubitus of the patients, the quality of images was poor. To increase the accuracy of diagnosis, a posterior thoracic view through the pleural effusion in the sitting position was used. This view allowed the diagnosis of mixed aortic valve disease (aortic stenosis and regurgitation) and the quantification of valve disease through multiparametric criteria as recommended by current guidelines. The posterior thoracic view, when feasible, may provide a useful option in the assessment of cardiac structures and further diagnostic information in technically difficult echocardiographic examinations.

## Introduction

### Case Presentation

**FIGURE 1:**
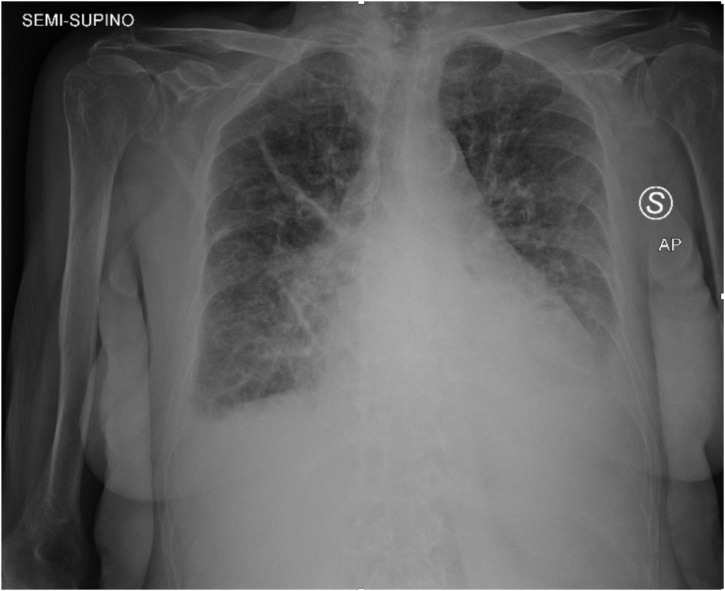
Chest X-ray was acquired in the semi-sitting position and showed bilateral diffuse pulmonary infiltrates consistent with pulmonary edema and bilateral pleural effusion.

**FIGURE 2:**
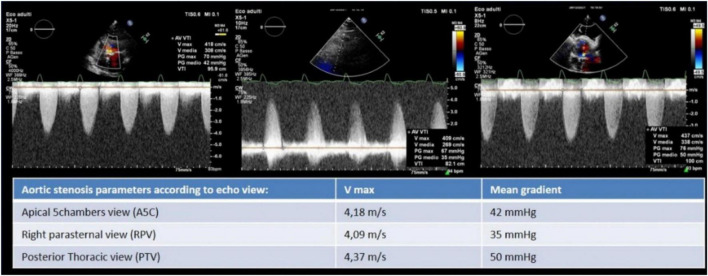
Aortic stenosis severity evaluation by Vmax and mean gradient from apical five chambers, right parasternal, and posterior thoracic view where the latter allowed to record the highest Vmax and mean gradient.

**FIGURE 3:**
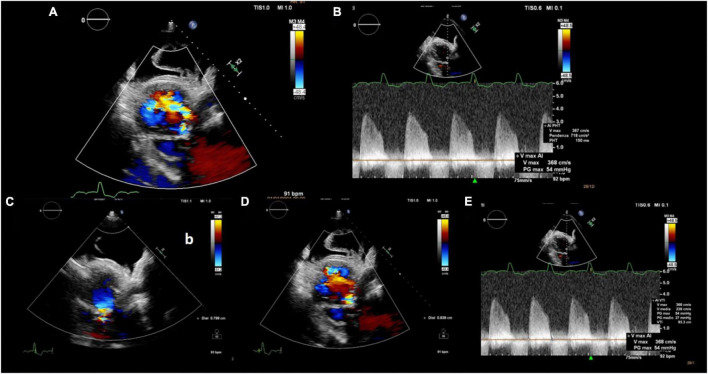
Multiparametric evaluation of aortic regurgitation severity: **(A)** Wide color flow regurgitant jet area, **(B)** PHT 150 ms, **(C)** PISA radius 8 mm, **(D)** vena contracta 8 mm, and **(E)** aortic regurgitation VTI 93 cm and Vmax 3.7 cm/s resulting in an EROA 0.52 cm^2^ and regurgitant volume 50 ml. PHT: pressure half time; PISA: proximal isovelocity surface area; VTI: velocity time integral; EROA: effective regurgitant orifice area.

An 85-year-old woman was admitted to our institution with acute respiratory distress. Clinical examination revealed a 5/6 systo-diastolic murmur radiating to both carotid arteries and bilateral lung crackles. Laboratory workup was notable for a N-terminal pro-brain natriuretic peptide (NT-proBNP) of 15,000 ng/L (normal range: < 300 ng/L) and a moderate high-sensitive troponin T elevation of 245 ng/L (normal range: < 14 ng/L) with no increase on serial testing. The hemoglobin and renal function were normal. Evaluation by chest X-ray showed bilateral diffuse pulmonary infiltrates consistent with pulmonary edema and bilateral pleural effusion (Figure 1). The patient was admitted to CCU. A transthoracic bedside echocardiogram (with EPIQ 7 C, X5-1, Philips Healthcare) was performed, but it was extremely difficult to obtain good quality images from standard views given the orthopneic decubitus of the patient. However, the aortic valve was heavily calcified, and severe aortic stenosis was documented with a mean gradient of 42 mmHg from the apical 5 chambers view and 35 mmHg from the right parasternal view, with a functional aortic valve area ≈0.6 cm^2^ (Figure 2). The left ventricle showed eccentric hypertrophy with a moderate diffuse reduction of ejection fraction (EF 35–40%). Then, the patient was positioned sitting upright, because the left pleural effusion offered an additional acoustic window—the posterior thoracic window (PTW)—that allowed better alignment of the ultrasound beam with the aortic jet. Mean gradient was recorded definitely higher than that from the standard view at 50 mm Hg (Figure 2). Moreover, aortic regurgitation that appeared mild from the standard view was documented as significant from PTW (Figure 3). In fact, a proper alignment of the aortic regurgitation jet was feasible from this view with a quantification of severity with multiparameter criteria as suggested by the guidelines (pressure half time 150 ms, vena contracta 8 mm, effective regurgitant orifice area 0.5cm^2^, regurgitant volume 50 ml; Figure 3).

Therefore, the final diagnosis was symptomatic severe mixed aortic valve disease with severe stenosis and severe regurgitation, associated with acute decompensated heart failure. The patient was stabilized by medical therapy and then addressed to transaortic valve replacement (TAVI). At the 6-month follow-up, the patient was asymptomatic with a functional capacity expected for her age.

## Discussion

Obtaining adequate echocardiographic images in critically ill patients is important for accurate diagnosis and treatment. For several reasons, this group of patients remains among the most challenging with regard to the quality of echocardiographic images.

**FIGURE 4:**
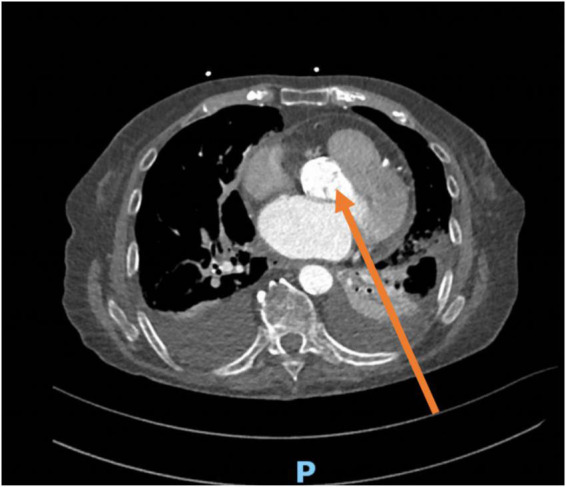
CT scan from the same patient showing the alignment of the Doppler beam in the posterior thoracic view toward the aortic valve (arrow). The transducer is positioned between posterior intercostal spaces, parallel to the ribs.

In the presence of pleural effusion, many cardiac structures may be evaluated from PTW (1), also known as subscapular retro cardiac imaging (2). Normally, imaging through the posterior thoracic window is not possible as the lungs overlay the heart and the ultrasound beam is reflected at the tissue-air interface (3), while the presence of a large left pleural effusion minimizes the impedance to ultrasound transmission (4). This view is excellent to adequately assess the native and prosthetic aortic valve velocity/gradients due to the parallel alignment between the ultrasound beam, left ventricular outflow tract, and aortic root (3) (Figure 4). Indeed, it is now well established that the “Doppler intercept angle,” the angle between the ultrasound beam and aortic jet, may strongly influence the hemodynamic assessment of aortic valve stenosis (5). This explains the reason why guidelines not only recommend the use of apical views but also advocate for multiple transducer positions to obtain the most accurate peak velocities across a stenotic aortic valve (6).

Recently, Benfari et al. demonstrated that the right parasternal view is highly feasible and can be crucial to properly assess aortic valve stenosis severity (avoiding misalignment), thereby resolving some inconsistencies between mean gradient and aortic valve area; however, this view is still not routinely used in clinical practice (7, 8).

To the best of our knowledge, only few cases (9) and two case series have been published using PTW in the assessment of native or prosthetic aortic valve (3, 10), but none of them reported a mixed (stenosis and regurgitation) aortic valve disease and the major impact of the PTW in the assessment of both stenosis and regurgitation. Indeed, the present case is unique because PTW allowed to correctly diagnose and quantify not only the highest velocity of aortic stenosis compared with other echocardiographic views but also aortic regurgitation with multiple parameters.

In conclusion, in the presence of pleural effusion and technically difficult echocardiographic examinations, the PTW should always be considered in the assessment of cardiac structures, including the aortic valve as a potentially useful option to provide accurate diagnostic information.

## Data Availability Statement

The original contributions presented in this study are included in the article/supplementary material, further inquiries can be directed to the corresponding author.

## Ethics Statement

Ethical review and approval was not required for the study on human participants in accordance with the local legislation and institutional requirements. The patients/participants provided their written informed consent to participate in this study. Written informed consent was obtained from the participant for the publication of this case report. Written informed consent was obtained from the individual(s) for the publication of any potentially identifiable images or data included in this article.

## Author Contributions

All authors listed have made a substantial, direct, and intellectual contribution to the work, and approved it for publication.

## Conflict of Interest

The authors declare that the research was conducted in the absence of any commercial or financial relationships that could be construed as a potential conflict of interest.

## Publisher’s Note

All claims expressed in this article are solely those of the authors and do not necessarily represent those of their affiliated organizations, or those of the publisher, the editors and the reviewers. Any product that may be evaluated in this article, or claim that may be made by its manufacturer, is not guaranteed or endorsed by the publisher.
